# Contralateral Adrenal Metastasis from Clear Cell Renal Cell Carcinoma After Right Radical Nephrectomy and Caval Thrombectomy: A Case Report and Review of the Literature

**DOI:** 10.7759/cureus.99951

**Published:** 2025-12-23

**Authors:** Luis Fernando Aguilar-Urrea, Hector Alejandro Cardenas-Alvarez, Samantha B Medrano-Juarez, Alejandro Hernández-Gutiérrez, Patricio Marcelo Quintanilla-Trevillo, Edwards Alejandro Rodríguez-Hinojosa, Raquel Garza-Guajardo, Adrian Gutierrez-Gonzalez, Pedro A. Madero-Morales

**Affiliations:** 1 Urology, Hospital Universitario Dr. José Eleuterio Gonzalez Universidad Autonóma de Nuevo Leon, Monterrey, MEX; 2 Pathology, Hospital Universitario Dr. José Eleuterio Gonzalez Universidad Autonóma de Nuevo Leon, Monterrey, MEX

**Keywords:** case report, clear cell renal cell carcinoma, contralateral adrenal metastasis, laparoscopic adrenalectomy, oligometastatic disease, review of the literature

## Abstract

Contralateral adrenal metastasis from clear cell renal cell carcinoma (ccRCC) is considered an exceptionally uncommon event. Early detection is crucial for optimal management, especially in high-grade tumors with vascular invasion. We report a 50-year-old woman with type 2 diabetes mellitus and obesity (BMI 29) who underwent right radical nephrectomy and inferior vena cava thrombectomy for pT3bpNxMx ccRCC. One year postoperatively, imaging revealed a solitary metachronous metastasis in the left adrenal gland, which progressively enlarged despite being under systemic therapy with pembrolizumab. A laparoscopic transabdominal adrenalectomy was successfully performed, and histopathology confirmed metastatic ccRCC. Postoperative recovery was uneventful, with adequate endocrine management ensuring normal adrenal function. A review of literature suggests that contralateral adrenal metastasis at presentation is rare and usually associated with large, high-grade primary tumors. The mechanism may involve hematogenous spread through the renal and adrenal venous systems. Surgical resection remains the mainstay of treatment, offering the best outcomes in selected patients without extra-adrenal disease. Long-term oncologic surveillance is essential in patients with ccRCC. In oligometastatic disease, adrenalectomy can offer significant diagnostic and therapeutic value. A multidisciplinary approach is crucial for optimizing outcomes.

## Introduction

Renal cell carcinoma (RCC) accounts for approximately 2-3% of all adult malignancies, with the clear cell subtype responsible for about 75-85% of cases [1,2]. Although the most common metastatic sites include the lungs, bones, liver, and brain, solitary metastasis to the contralateral adrenal gland is exceptionally rare, with an incidence of less than 1% in large series [1,3,4]. In such scenarios, metastasis may occur either synchronously or metachronously, and its detection is often incidental during routine oncologic follow-up [5-7].

Several case reports and small series have documented this rare pattern of contralateral adrenal metastasis in patients with RCC [1,2,5]. These cases become even more complex when the primary tumor exhibits vascular invasion or tumor thrombus formation, as seen in pT3b disease [8,9]. Although systemic therapies, including immune checkpoint inhibitors such as pembrolizumab, have shown efficacy in metastatic RCC, surgery remains a cornerstone in the management of carefully selected oligometastatic patients [10-15].

Herein, we report a rare case of a 50-year-old woman with right-sided clear cell renal cell carcinoma (ccRCC) who underwent radical nephrectomy with caval thrombectomy and subsequently developed a solitary metachronous metastasis to the contralateral adrenal gland. The metastatic lesion was successfully managed with laparoscopic adrenalectomy, and histopathological examination confirmed metastatic ccRCC. A concise review of the literature is included to highlight the diagnostic considerations and therapeutic strategies for this uncommon clinical presentation.

## Case presentation

A 50-year-old woman with a history of type 2 diabetes mellitus and obesity (BMI 29 kg/m²) was referred to our center in September 2021 after abdominal imaging revealed a right renal mass with cortical invasion measuring 112 × 93 mm and irregularity of the right adrenal gland. Follow-up computed tomography (CT) confirmed the renal tumor. It identified a tumor thrombus in the infrahepatic inferior vena cava (IVC), which was a level two tumor thrombus, according to the Neves Zincke classification [15]. She was scheduled for a right radical nephrectomy with IVC thrombectomy.

In January 2022, the patient underwent right radical nephrectomy, IVC thrombectomy with cavoplasty, and cholecystectomy for a grade II gallbladder lesion. The total operative time was four hours, with an estimated blood loss of 3,000 mL, and 1,000 mL of packed red blood cells were transfused. She was admitted to the intensive care unit (ICU) for four days and discharged on postoperative day six.

Histopathological examination revealed ccRCC, International Society of Urological Pathology (ISUP) grade III [16], without sarcomatoid or rhabdoid features. The tumor infiltrated but did not penetrate the renal capsule and was less than 0.1 cm from the fascia. Surgical margins were positive for vascular invasion, and the right adrenal gland was unremarkable. The IVC thrombus consisted of fragmented ccRCC tissue. Pathological staging was reported as pT3b, pNx, pMx.

**Figure 1:**
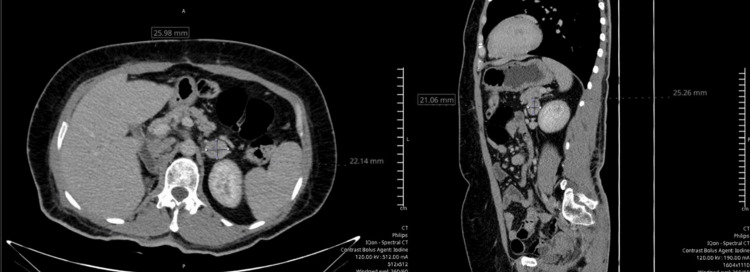
Contrast-enhanced CT demonstrating progressive left adrenal lesion Contrast-enhanced CT scan of the left adrenal gland (November 2023) demonstrating a lesion measuring 25 × 21 mm with central necrosis and 28% contrast washout. Compared with prior imaging in June 2023 (18 × 10 mm), the lesion shows progressive growth despite systemic therapy. Left panel: axial view. Right panel: sagittal view.

In March 2022, the patient was clinically staged as stage III (pT3b, pNx, Mx, G3), and adjuvant pembrolizumab (200 mg every three weeks) was initiated for one year. In May 2022, imaging revealed a 13 × 10 mm lesion in the left adrenal gland with arterial enhancement (136 HU) and venous washout, suggestive of metastasis, while no local recurrence was observed at the surgical site. By August 2022, the lesion had increased to 18 × 10 mm. Pembrolizumab was discontinued in June 2023 after the seventh cycle due to cardiotoxicity. A CT scan performed in June 2023 showed no significant change in the left adrenal gland, whereas follow-up imaging in November 2023 (Figure 1) demonstrated growth of the lesion to 25 × 21 x 25 mm with central necrosis and 28% washout. 

A percutaneous biopsy was proposed in December 2023 and performed in April 2024, but the sample was non-diagnostic due to insufficient tissue. By May 2024, a control CT scan revealed a heterogeneous left adrenal lesion with areas of necrosis and post-contrast enhancement (76 HU), measuring approximately 48 × 44 × 49 mm (previously 25 × 21 × 25 mm). The case was discussed in a multidisciplinary tumor board involving Urology, Oncology, RadioOncology and Imaging in August 2024, and laparoscopic adrenalectomy was recommended.

Preoperative laboratory tests of serum sodium, potassium, and chloride were within normal limits. Random urinary electrolytes were also normal. A biochemical evaluation was performed, ruling out endocrine functionality. Urinary metanephrines were not measurable. Endocrinology was included due to the transoperative implications regarding the anesthesiological management for adrenal insufficiency. On August 14, 2024, additional laboratory tests for glucose, creatinine, blood urea nitrogen (BUN), hemoglobin, hematocrit, and platelets were also done (Table 1).

**Table 1:** Summary of preoperative laboratory investigations Laboratory parameters remained within normal limits, except for a mild elevation in glucose and blood urea nitrogen (BUN). The biochemical profile ruled out endocrine activity of the adrenal lesion.

Parameter	Patient value	Reference range
Serum sodium	137.2 mEq/L	135–145 mEq/L
Serum potassium	4.2 mEq/L	3.5–5.1 mEq/L
Serum chloride	101.2 mEq/L	98–107 mEq/L
Urinary sodium	54 mEq/L	54-190 mEq/L
Urinary potassium	19.7 mEq/L	20-80 mEq/L
Urinary chloride	29 mEq/L	75-199 mEq/L
Glucose	138 mg/dL	70–110 mg/dL
Creatinine	0.81 mg/dL	0.6–1.2 mg/dL
Blood urea nitrogen (BUN)	27.5 mg/dL	7–20 mg/dL
Estimated GFR (eGFR)	87 mL/min/1.73 m²	≥90 mL/min/1.73 m²
Hemoglobin	15.3 g/dL	12–16 g/dL
Hematocrit	46%	36–46%
Platelets	250,000/ mm³	150,000–400,000/mm³

Insulin glargine was increased to 36 IU, and metformin 850 mg once daily was added.

**Figure 2:**
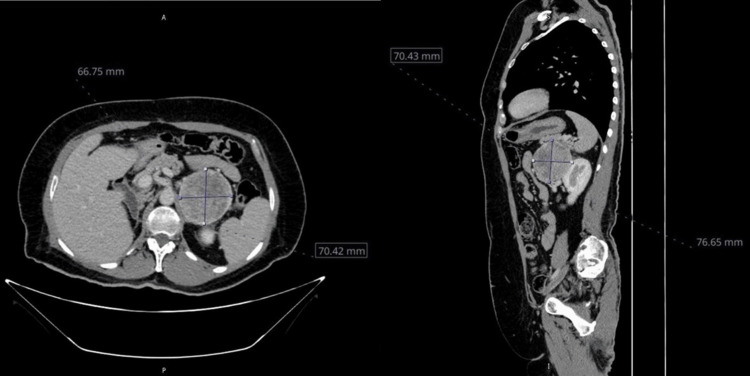
Progressive enlargement of the left adrenal lesion on contrast-enhanced CT Contrast-enhanced CT scan of the left adrenal gland (February 2025) showing further enlargement of the lesion to 70 × 76 × 70 mm. The lesion demonstrates internal necrosis and post-contrast enhancement (69 HU in the venous phase, 62 HU in the delayed phase). No additional metastatic lesions were identified. Left panel: coronal view. Right panel: sagittal view.

During the follow-up in February 2025, a control CT demonstrated further enlargement of the left adrenal gland to 70 × 76 × 70 mm (previously 48 × 44 × 49 mm), with post-contrast enhancement (69 HU in the venous phase and 62 HU in the delayed phase) and internal necrosis. No pulmonary, hepatic, splenic, or nodal metastases were identified (Figure 2). 

A transabdominal laparoscopic left adrenalectomy was performed in June 2025. The procedure was uneventful, with an estimated blood loss of 350 mL and a postoperative hospital stay of four days.

**Figure 3:**
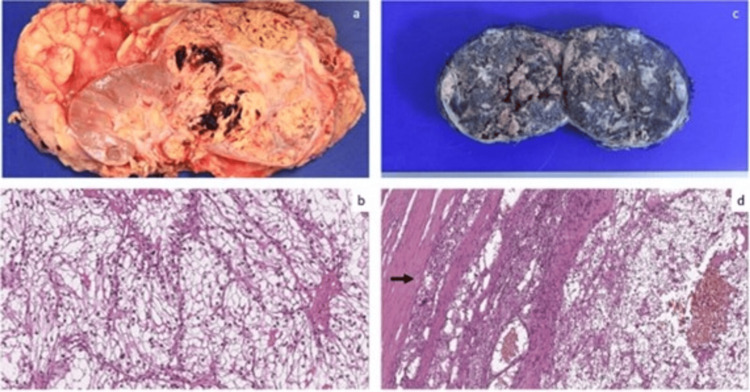
Pathologic features of primary clear cell renal cell carcinoma (ccRCC) and adrenal metastasis (a) Right renal tumor measuring 10.5 × 10 cm, yellowish, with areas of hemorrhage; it infiltrates the capsule and is very close to Gerota’s fascia; (b) Clear cell carcinoma, nuclear grade 3, arranged in nests with a prominent capillary network (hematoxylin and eosin stain, ×220); (c) Section of the left adrenal gland showing a tumor measuring 10 cm in greatest diameter with extensive necrosis and hemorrhage; (d) Histologic section showing scant residual adrenal tissue (arrow) and ccRCC with hemorrhage (hematoxylin and eosin stain, ×100).

Pathological analysis confirmed metastatic ccRCC in the left adrenal gland, measuring 10 cm with extensive tumor necrosis (Figure 3).

During postoperative follow-up, the patient demonstrated excellent clinical recovery 10 days after surgery, with stable glycemic control and appropriate management of adrenal insufficiency under endocrinology supervision.

## Discussion

Contralateral solitary adrenal metastasis from ccRCC is rare, with an incidence of less than 1% [1,2,5]. It typically appears years after primary surgery and remains asymptomatic, underscoring the importance of long-term imaging surveillance. In this case, a metachronous adrenal metastasis was identified five months after radical nephrectomy and caval thrombectomy. The aggressive nature of pT3b ccRCC with vascular invasion and positive surgical margins likely contributed to early dissemination despite the absence of initial metastatic disease. The initial biopsy was non-diagnostic, consistent with literature reporting the limited accuracy of fine-needle aspiration for adrenal lesions [17-19]. Radiologic findings such as arterial enhancement and delayed washout should raise suspicion for metastasis in high-risk patients.

Although pembrolizumab was initiated as adjuvant therapy, it was discontinued due to cardiotoxicity. Evidence suggests that surgical metastasectomy provides a survival benefit in selected patients with oligometastatic, completely resectable disease [10,12-14]. In this case, laparoscopic adrenalectomy was successfully performed with minimal morbidity, confirming the diagnosis. This report supports existing evidence that, in carefully selected patients with solitary contralateral adrenal metastasis, surgery may serve both diagnostic and therapeutic purposes [1-3,10-14], emphasizing the importance of a multidisciplinary and individualized approach in complex RCC cases [20-26].

Comparison with previously reported cases

Several reports have described solitary contralateral adrenal metastasis from RCC, emphasizing its potential for late recurrence even after many years of disease-free survival. Including the present case, we identified 10 solitary cases of contralateral adrenal metastasis, which are summarized in Table 2.

**Table 2:** Summary of reported cases of contralateral adrenal metastasis from renal cell carcinoma

Author (Year)	Age / Sex	Primary tumor side	Time to adrenal metastasis	Laterality	Symptoms at diagnosis	Imaging modality	Treatment performed	Pathology confirmation	Outcome / Follow-up
Doyya et al. (2025) [8]	57 / Female	Left	2 months	Contralateral	Hematuria	CT	Adrenalectomy	Yes	6 months follow-up
Öğreden et al. (2023) [23]	52 / Male	Right	6 months	Contralateral	Asymptomatic	MRI	Adrenalectomy	Yes	6 months follow-up
Paiva et al. (2023) [24]	55 / Male	Left	Synchronous	Contralateral	Pain	CT	Adrenalectomy	Yes	Alive
Ahmed et al. (2019) [20]	57 / Female	Left	15 years	Contralateral	Asymptomatic	CT	Adrenalectomy	Yes	2 years follow-up
Kaneko et al. (2019) [25]	71 / Male	Left	2 years	Contralateral	Asymptomatic	CT	Adrenalectomy	Yes	9 months follow-up
Murtaza et al. (2017) [6]	38 / Male	Left	Synchronous	Contralateral	Pain	CT	Adrenalectomy	Yes	Alive
Piotrowicz et al. (2015) [26]	47 / Female	Left	18 years	Contralateral	Asymptomatic	CT	Adrenalectomy	Yes	Alive
Balasar et al. (2015) [2]	58 / Female	Right	5 years	Contralateral	Asymptomatic	CT	Adrenalectomy	Yes	Alive
Ozturk et al. (2014) [11]	68 / Male	Left	8 years	Contralateral	Asymptomatic	CT	Adrenalectomy	Yes	Alive
Utsumi et al. (2008) [14]	64 / Male	Left	Synchronous	Contralateral	Pain	MRI	Adrenalectomy	Yes	6 months follow-up

The mean age of affected patients was approximately 57 years, with a slight male predominance. The time to adrenal recurrence varied widely, ranging from synchronous presentation to 18 years after nephrectomy, highlighting the indolent and unpredictable behavior of ccRCC. 

Most patients were asymptomatic at the time of detection, with the diagnosis established incidentally during radiological surveillance. CT was the most frequently used imaging modality, while magnetic resonance imaging (MRI) and positron emission tomography (PET/CT) were occasionally employed to confirm the adrenal origin of the lesion and exclude other sites of metastasis. Imaging characteristics such as arterial phase enhancement and delayed washout may suggest metastatic RCC, although definitive diagnosis requires histopathological confirmation [6,8,17-26].

Surgical metastasectomy remains the mainstay of treatment for solitary adrenal metastases in appropriately selected patients. In our review, all reported patients underwent adrenalectomy, predominantly through minimally invasive approaches, with favorable short-term outcomes. Adrenalectomy offers both diagnostic and therapeutic benefits, potentially prolonging survival in oligometastatic disease when systemic burden is limited [6-9]. Advances in systemic therapy, including immune checkpoint inhibitors and tyrosine kinase inhibitors, have improved outcomes in metastatic RCC; however, surgery continues to play a key role in the management of isolated metastatic lesions in fit patients [10-12].

Given the variable latency period and the possibility of late solitary metastasis, long-term follow-up is essential in patients with high-grade or vascular-invasive RCC. Multidisciplinary evaluation, including urologic oncology, radiology, and endocrinology, is critical for individualized management. Our case reinforces the importance of continued vigilance, as timely detection and resection of solitary contralateral adrenal metastasis can lead to excellent oncologic outcomes.

## Conclusions

Contralateral adrenal metastasis from ccRCC is a rare but clinically relevant manifestation that may occur even shortly after radical nephrectomy, particularly in patients with aggressive pathological features such as vascular invasion and high tumor grade. This case highlights the importance of strict and prolonged oncologic surveillance in patients with high-risk ccRCC, as adrenal metastases are frequently asymptomatic and detected incidentally during follow-up imaging. Progressive growth, arterial enhancement, and limited contrast washout on imaging should raise suspicion for metastatic disease, especially when biopsy results are inconclusive.

In selected patients with isolated or oligometastatic disease, surgical adrenalectomy remains a valuable diagnostic and therapeutic option, even in the era of modern systemic therapies. Minimally invasive adrenalectomy can be performed safely with low morbidity and may contribute to durable disease control when complete resection is achievable. Optimal management of these complex cases requires a multidisciplinary approach involving urology, oncology, radiology, and endocrinology to individualize treatment strategies and ensure appropriate perioperative care. This report supports the role of metastasectomy as part of a comprehensive treatment plan in carefully selected patients with metastatic ccRCC.
